# A DNA vaccine for Crimean-Congo hemorrhagic fever protects against disease and death in two lethal mouse models

**DOI:** 10.1371/journal.pntd.0005908

**Published:** 2017-09-18

**Authors:** Aura R. Garrison, Charles J. Shoemaker, Joseph W. Golden, Collin J. Fitzpatrick, John J. Suschak, Michelle J. Richards, Catherine V. Badger, Carolyn M. Six, Jacqueline D. Martin, Drew Hannaman, Marko Zivcec, Eric Bergeron, Jeffrey W. Koehler, Connie S. Schmaljohn

**Affiliations:** 1 Virology Division, United States Army Medical Research Institute of Infectious Diseases, Fort Detrick, Maryland, United States of America; 2 Ichor Medical Systems, Inc., San Diego, California, United States of America; 3 Viral Special Pathogens Branch, National Center for Emerging and Zoonotic Infectious Diseases, Centers for Disease Control and Prevention, Atlanta, Georgia, United States of America; 4 Diagnostics Systems Division, United States Army Medical Research Institute of Infectious Diseases, Fort Detrick, Maryland, United States of America; 5 Headquarters Division, United States Army Medical Research Institute of Infectious Diseases, Fort Detrick, Maryland, United States of America; University of Thessaloniki, Greece, UNITED STATES

## Abstract

Crimean-Congo hemorrhagic fever virus (CCHFV) is a tick-borne virus capable of causing a severe hemorrhagic fever disease in humans. There are currently no licensed vaccines to prevent CCHFV-associated disease. We developed a DNA vaccine expressing the M-segment glycoprotein precursor gene of CCHFV and assessed its immunogenicity and protective efficacy in two lethal mouse models of disease: type I interferon receptor knockout (IFNAR^-/-^) mice; and a novel transiently immune suppressed (IS) mouse model. Vaccination of mice by muscle electroporation of the M-segment DNA vaccine elicited strong antigen-specific humoral immune responses with neutralizing titers after three vaccinations in both IFNAR^-/-^ and IS mouse models. To compare the protective efficacy of the vaccine in the two models, groups of vaccinated mice (7–10 per group) were intraperitoneally (IP) challenged with a lethal dose of CCHFV strain IbAr 10200. Weight loss was markedly reduced in CCHFV DNA-vaccinated mice as compared to controls. Furthermore, whereas all vector-control vaccinated mice succumbed to disease by day 5, the DNA vaccine protected >60% of the animals from lethal disease. Mice from both models developed comparable levels of antibodies, but the IS mice had a more balanced Th1/Th2 response to vaccination. There were no statistical differences in the protective efficacies of the vaccine in the two models. Our results provide the first comparison of these two mouse models for assessing a vaccine against CCHFV and offer supportive data indicating that a DNA vaccine expressing the glycoprotein genes of CCHFV elicits protective immunity against CCHFV.

## Author summary

Crimean-Congo hemorrhagic Fever Virus (CCHFV) is a tick-borne virus capable of causing lethal human disease against which there are currently no approved vaccines. In this study, we compared the immunogenicity and protective efficacy of a candidate DNA vaccine expressing the glycoprotein precursor gene of CCHFV in two mouse models. In addition to the recently established IFNAR^-/-^ mouse pathogenesis model, we also tested the vaccine in a novel murine system in which the interferon (IFN) α/β signaling response of immunocompetent mice is transiently suppressed. We found that the DNA vaccine elicited high humoral immune responses and provided significant protection against challenge with CCHFV in both mouse models. These findings further our understanding of the requirements for a CCHFV vaccine and provide a new mouse model for the development of CCHFV countermeasures.

## Introduction

Crimean-Congo hemorrhagic fever virus (CCHFV) is a tick-borne virus with a wide geographical distribution, including Africa, the Balkans, the Middle East, Russia and western Asia [1]. CCHFV, a member of the *Nairoviridae* family in the *Bunyavirales* order, has a tripartite, negative-sense RNA genome comprising small (S), medium (M) and large (L) segments. The S segment encodes the nucleocapsid protein (N), the M segment encodes the glycoprotein open reading frame (ORF) that is cleaved into two structural glycoproteins (G_N_ and G_C_) and non-structural proteins, and the L segment encodes the RNA-dependent RNA polymerase (reviewed in [2]). CCHFV infection can cause Crimean-Congo hemorrhagic fever (CCHF), a severe, often fatal, human disease characterized by hemorrhage. Humans appear to be uniquely affected by CCHFV as infection in other animals, including agricultural animals, does not cause significant disease and the virus is generally cleared after a brief period of viremia [3], (reviewed in [4]). Human infection can result from the bite of infected ticks, as well as from exposure to infected agricultural animals during butchering [5]. Nosocomial CCHFV infections primarily impacting medical staff have also been reported [6, 7]. Between 1953 and 2010, the prevalence and geographical distribution of CCHFV has been increasing with mortality rates ranging from 5–67%, and from 2002 to 2016 more than 9700 CCHF patients were reported in Turkey alone [5, 8–10]. There is also some evidence that the range of CCHFV is expanding, as CCHFV infected ticks were found in Spain in 2010 and the first reported human infections in Southwestern Europe occurred in Spain in 2016 [11, 12]. As of 2017, CCHFV has been designated as one of ten priority emerging infectious diseases by the World Health Organization. This has led to an increased awareness of the need for medical countermeasures aimed at preventing this disease.

To date, the only CCHFV vaccine tested in humans is a formalin inactivated, suckling mouse brain-derived, virus preparation formulated with an aluminum hydroxide adjuvant, which was developed in Bulgaria [13]. Evaluation of this vaccine in healthy human volunteers showed that four vaccinations elicited high levels of total IgG but only low levels of neutralizing antibodies [14]. Individuals vaccinated four times were also found to have T-cell responses to N that were approximately ten-fold higher than those individuals receiving a single vaccination. The historical absence of a lethal animal model of CCHF has precluded laboratory evaluation of the efficacy of this vaccine, and controlled human studies have not been reported.

Although CCHFV is apathogenic in wild-type mice, two lethal mouse models, a STAT-1 knockout mouse model (C57BL/6 background) and interferon α/β (IFN-α/β) receptor 1 knockout (IFNAR^-/-^) mouse models (C57BL/6 or A129 background), have been developed, which recapitulate some of the clinical features of CCHF in humans, including severe hepatic injury [15–17]. Both of these mouse systems have been used to evaluate CCHFV vaccines. A study in the STAT-1 mouse model showed that a CCHFV subunit vaccine could elicit strong neutralizing antibodies; however, the mice were not protected from lethal disease, indicating that the STAT-1 model, which has defects in both type I and II interferon signaling systems, is perhaps too sensitive to CCHFV for vaccine evaluation [18, 19]. In contrast, experimental CCHFV vaccines have recently been reported to show protective efficacy in the IFNAR^-/-^ (A129) model [20, 21]. This includes a formalin-inactivated CCHFV (cell culture-derived Turkey-Kelkit06 strain) vaccine that demonstrated protective efficacy in IFNAR^-/-^ (A129) against a lethal infection with the homologous strain of CCHFV. Additionally, a modified vaccinia Ankara (MVA)-vectored vaccine expressing the CCHFV M-segment ORF (MVA-GP), from the IbAr 10200 strain, provided complete protection from lethal infection with the homologous strain of CCHFV [20, 22]. Investigation of vaccine-induced immune responses with the MVA-GP vaccine suggested that both the cellular and humoral arms were critical for protective efficacy. A CCHFV DNA vaccine comprised of three separate plasmids encoding G_N_, G_C_, and N, each tethered to a ubiquitin coding sequence, was also shown to elicit protective immunity in IFNAR^-/-^ A129 mice [23].

We previously developed a CCHFV DNA vaccine encoding the CCHFV M-segment ORF that induced neutralizing antibodies in mice when delivered by gene gun, albeit inconsistently [24]. Efficacy testing was not possible at the time due to the lack of a lethal animal model for CCHFV. Here, we report the improvement of this DNA vaccine by gene optimization of the full length M segment. We evaluated the immunogenicity and protective efficacy of this optimized vaccine when delivered by intramuscular electroporation (IM-EP) in two lethal CCHFV models, IFNAR^-/-^ (C57BL/6) mice and a novel transiently immune-suppressed (IS) C57BL/6 mouse CCHFV model. The IS mouse model exploits a monoclonal antibody (MAb-5A3) that blocks signaling via the IFNAR-1 subunit of the murine IFN α/β receptor. This transient IFN blockade has been used in several other viral studies to examine the role of type I IFN in disease [25–27]. The advantage of the transient IFN-α/β blockade model is that vaccines can be evaluated in mice with intact IFN-α/β signaling, and then during challenge IFN- α/β can be blocked to test protective efficacy. To our knowledge, this is the first direct comparison of the IFNAR^-/-^ and IS mouse model for assessing the immunogenicity and efficacy of a CCHFV vaccine.

## Materials and methods

### Ethics statement

This work was supported by an approved USAMRIID IACUC animal research protocol. Research was conducted under a USAMRIID IACUC supported and approved protocol in compliance with the Animal Welfare Act, PHS Policy, and other Federal statutes and regulations relating to animals and experiments involving animals. The facility where this research was conducted is accredited by the Association for Assessment and Accreditation of Laboratory Animal Care, International and adheres to principles stated in the Guide for the Care and Use of Laboratory Animals, National Research Council, 2011 [28]. This research was conducted at a facility that is accredited by the Association for Assessment and Accreditation of Laboratory Animal Care International (AAALAC). Humane endpoints were used during these studies, and mice that were moribund, according to an endpoint score sheet, were humanely euthanized. Mice were euthanized by CO_2_ exposure using compressed CO_2_ gas followed by cervical dislocation. However, even with multiple observations per day, some animals died as a direct result of the infection.

### Cells and virus

Hep G2 cells were propagated in Modified Eagle’s Medium with Earle’s Salts (MEM) (Corning) supplemented with 10% fetal bovine serum (FBS) (Gibco), and 1X Glutamax (Gibco). BHK-21 cells were cultured in Dulbecco’s Modified Eagle’s Medium (DMEM) (Corning) supplemented with: 10% heat-inactivated FBS, 1% penicillin/streptomycin (Gibco), 1% sodium pyruvate (Sigma), and 1% L-glutamine (HyClone). SW-13 cells were cultured in DMEM supplemented with 10% FBS, 1% penicillin/streptomycin, 1% HEPES (Sigma), 1% non-essential amino acids (Gibco), and 1% L-glutamine. COS-7 cells were propagated in MEM supplemented with 10% heat-inactivated FBS, 1% penicillin/streptomycin, and 1% L-glutamine. All cells were maintained at 37°C/5% CO_2_. CCHFV strain IbAr 10200 (USAMRIID collection) was used for all experiments. This virus was previously passaged nine times in suckling mouse brain and then propagated three times in Hep G2 cells. The virus was collected from clarified cell culture supernatants and stored at -80°C. All CCHFV work was performed in BSL-4 containment.

### DNA vaccine construction

The M-segment ORF of strain IbAr 10200 (Accession # AAA86616) was optimized by GeneArt for human codon usage and deletion of known motifs that are detrimental to mRNA stability or expression. The optimized gene was *de novo* synthesized and cloned into pCAGGS (a generous gift from Robert Doms, University of Pennsylvania). The codon-optimized M-segment ORF was subcloned into the mammalian expression vector pWRG7077 at the NotI sites to create the optimized CCHFV-M DNA vaccine [24]. Nucleotide sequences were confirmed prior to vaccination.

### Flow cytometry

COS-7 cells were propagated in 12-well tissue culture plates (Corning) to 70–90% confluency in MEM. The medium in these plates was replaced with an Opti-MEM (Gibco) solution containing 2% FBS and 0.1% Gentamicin (Sigma) (cOpti-MEM). The DNA plasmids were transfected into COS-7 cells in a dilution series of 250, 100, 50, or 0 ng in duplicate using FuGENE 6 (Promega) according to manufacturer’s directions. Transfected cells were incubated for 44 h, washed once with PBS and then detached by adding 100 μL of trypsin-EDTA (Gibco) per well and incubating at ambient temperature. The cells were washed three times in FACS buffer solution, PBS with 2% heat-inactivated FBS and 0.1% sodium azide (Sigma), fixed in Cytofix buffer (BD Biosciences) for 30 minutes at 4°C, and then washed once with FACS buffer as above. To detect the intracellular CCHFV glycoprotein, cells were permeabilized with Perm/Wash buffer (BD Biosciences) at ambient temperature for 15 minutes, and then centrifuged for five minutes at 980 x g, at 4°C. The permeabilized and non-permeabilized cells were incubated with 20 μg/ml of anti-CCHFV G_C_ mouse monoclonal antibody 11E7 (USAMRIID) in Perm/Wash buffer or PBS with 2% FBS respectively, and incubated for 30 minutes at 4°C. Cells were washed three times in Perm/Wash buffer and centrifuged for five minutes at 980 x g, at 4°C between each wash. Alexa Fluor 488-conjugated goat anti-rabbit IgG (Life Technologies) was diluted 1:200 and incubated with the cells for 20 minutes at 4°C, and then washed three times with FACS buffer. Cell pellets were re-suspended in 500 μl FACS buffer and analyzed on a FACSCalibur flow cytometer (BD Biosciences). Cells staining positive for intracellular glycoprotein were shown as a percentage of total cells per 10,000 events. Histograms and dot plots were generated using FlowJo flow cytometry analysis software (Tree Star Inc).

### Western blot

Following the aforementioned 44 h incubation post-transfection, COS-7 cells were lysed with 1X Protein Loading Buffer (LI-COR). The cell lysates were probe sonicated for 15–20 seconds each, mixed 9:1 with 2-mercaptoethanol (Sigma), and heated at 70°C for 10 min. Proteins were separated by SDS-PAGE in 10% Bis-Tris gels (NuPAGE) and transferred to polyvinylidene difluoride membranes (Invitrogen). The membranes were blocked with Odyssey Blocking Buffer in Tris–buffered Saline (TBS, LI-COR) and probed for either G_C_ with 4.1 μg/ml of monoclonal antibody 11E7 (USAMRIID) or G_N_ with 1:500 rabbit polyclonal anti-CCHFV G_N_ sera (a generous gift from Robert Doms, University of Pennsylvania) [29] prepared in TBS (Sigma) supplemented with 0.2% Tween-20 (TBST, Sigma) and incubated at 4°C overnight. The membranes were washed 3 times with TBST and incubated with IR680-conjugated anti-rabbit or IR800-conjugated anti-mouse secondary antibodies (LI-COR) diluted in TBST at ambient temperature for 1 hour. The membranes were washed an additional 3 times with TBST and imaged using an Odyssey CLx imaging system (LI-COR).

### DNA vaccination

Groups of 10 IFNAR^-/-^ or C57BL/6 mice were vaccinated in the anterior tibialis muscle with 25 μg of either the optimized CCHFV-M DNA vaccine (IbAr 10200) or the pWRG7077 empty vector using the Ichor TriGrid IM-EP system [30], under isoflurane anesthesia. All mice were vaccinated three times at three weeks intervals. Blood was obtained via submandibular bleeds prior to each vaccination.

### CCHF VLP production

Production of IbAr 10200 strain of CCHF VLPs (CCHF_VLP_) was performed with slight modification of methods reported previously [31]. Briefly, BHK-21 cells were propagated to 70–80% confluency in 10 cm^2^ round tissue culture plates and then transfected with 10 μg pC-M Opt (IbAr 10200), 4 μg pC-N, 2 μg L-Opt, 4 μg T7-Opt, and 1 μg Nano-luciferase encoding mini-genome plasmid using the Transit LT-1 (Mirus Bio) transfection reagent according to manufacturer’s instructions. Three days post-transfection, supernatants were harvested, cleared of debris, and VLPs were pelleted through a cushion of 20% sucrose in virus resuspension buffer (VRB; 130 mM NaCl, 20 mM HEPES, pH 7.4) by centrifugation for 2 h at 106,750 x g in an SW32 rotor at 4°C. VLPs were resuspended overnight in 1/200 volume VRB at 4°C, and then frozen at -80°C in single-use aliquots. Individual lots of CCHF_VLP_ were standardized by Western Blot analysis based on incorporation of N relative to a parallel gradient of recombinant N loaded on the same SDS-PAGE reducing gel. CCHF_VLP_ were also quantified using a TCID_50_ assay on SW-13 cells in 96-well, black-walled, clear-bottom plates (Corning). Plates were incubated with ten-fold dilutions of the CCHF_VLP_ overnight and were then processed for Nano Luciferase (Promega) expression. Wells that displayed a Nano Luciferase signal 3 standard deviations or greater above background levels were considered positive for VLP signal. VLP stock concentrations (TCID_50_ per mL) were calculated using the Reed and Muench formula [32].

### CCHF VLP neutralization assay

One day prior to the assay, 50,000 SW-13 cells were seeded into a 96-well black-walled, clear bottom tissue culture plate. All serum samples were heat inactivated at 56°C for 30 m. Half-log serial dilutions were made in duplicate from 1:25 to 1:25,368 and then an equal volume of medium with IbAr 10200 VLPs containing 237 TCID_50_ units was added and incubated at 37°C/5% CO_2_ for 1 h. Final effective dilutions of analyte sera ranged from 1:50 to 1:50,736. Half of this reaction mixture (50 μl) was then added to the previously aspirated target cell plate. Cells were incubated for 24 h before being lysed using NanoGlo Lysis buffer mixed with 1/50 dilution of NanoGlo substrate (Promega). Samples were mixed and incubated for 5 m at ambient temperature prior to the luminescent signal being measured on a Modulus Microplate Reader (Turner Biosystems) with an integration time of 5 s per well. To measure the effect of complement on neutralization, Low-Tox Guinea Pig Complement (Cederlane Labs) was reconstituted in DMEM, filtered, and added to the VLP/sera mixture at a final concentration of 5% and the assay was carried out as above. Data were analyzed as previously reported using GraphPad Prism software (GraphPad Software) [33].

### CCHF_VLP_ ELISA

High Bind ELISA plates (Corning) were coated overnight at 4°C with approximately 1 ng N equivalent of CCHF_VLP_ diluted in PBS per 96-well plate. The following day, plates were washed and then blocked with 3% goat serum/3% skim milk for 1 h at 37°C. All washes were done with PBS containing 0.2% Tween-20 (PBST, Sigma). Plates were washed again, prior to being loaded with two-fold serial dilutions of mouse sera in duplicate (dilution range 1:200 to 1:25,600). Serum dilutions were carried out in blocking buffer. Plates were incubated at ambient temperature for 1 h prior to being washed, and then incubated with a 1:1000 dilution of horse radish peroxidase (HRP) conjugated goat anti-mouse (SeraCare Inc.) in PBST for 1 h at ambient temperature. Plates were washed again and then developed with TMB substrate (SeraCare Inc.). Absorbance at the 450 nm wavelength was detected with a Tecan M1000 microplate reader. Pooled naïve sera collected prior to vaccination was used as an internal control on each assay group. A plate cutoff value was determined based on the average absorbance of the naïve control starting dilution plus 3 standard deviations. Only sample dilutions whose average was above this cut-off were registered as positive signal. Additional analysis was carried out using GraphPad Prism 6.

### Antibody isotype analysis

Plates were coated with CCHF_VLP_ as previously described. The following day, plates were washed and blocked, and two-fold serial dilutions of mouse sera starting at 1:100 were added to the wells of replicate plates. After 1 h incubation at ambient temperature, the plates were washed, and then incubated for 1 h with a 1:10,000 dilution of either anti-mouse IgG1 HRP conjugated antibody (Bethyl) or anti-mouse IgG2c HRP conjugated antibody (Bethyl). Plates were then washed, and TMB substrate was added and absorbance at 450 nm was recorded.

### Antibody avidity

Plates were coated with CCHF_VLP_. The following day, plates were washed, blocked and loaded with 1:200 dilutions of experimental sera for 1 h at ambient temperature. Plates were then washed before being exposed to concentrations of Sodium Thiocyanate (Sigma-Aldrich) ranging from 0–5.0 M. Samples were incubated for 15 m at ambient temperature before being washed. Secondary antibody incubation and development with TMB substrate was then performed as previously stated.

### Virus challenge and MAb-5A3 treatment

C57BL/6 mice were treated by the intraperitoneal (IP) route with MAb-5A3 (Leinco Technologies Inc) 24 h prior to (2.0 mg) and 24 h after (0.5mg) CCHFV challenge. IFNAR^-/-^ and IS C57BL/6 mice were challenged with 100 plaque forming units (PFU) of CCHFV strain IbAr 10200 by the IP route four weeks following the final vaccination. The mice were monitored daily for group weight changes, clinical score, and survival. Twenty-eight days following challenge the surviving mice were euthanized by exsanguination under deep anesthesia, and sera were collected for post-challenge analysis.

### CCHFV-N ELISA

N-reactive antibodies in challenged mice were detected by ELISA. Recombinant N was produced as previously reported [34] with minor modifications. Briefly, CCHFV N (strain IbAr 10200) was amplified and cloned into the vector pQE-30 (Qiagen) to have an N-terminal histidine tag. This insert was transferred into the plasmid pFastbac-1 (Invitrogen) in order to generate recombinant baculovirus according to manufacturer’s instructions using the Bac-to-Bac Baculovirus Expression System (Invitrogen). The recombinant CCHFV N protein was produced and purified by AI BioTech. Briefly, *Spodoptera frugiperda* (Sf9) insect cells were infected with the recombinant baculovirus and incubated at 28°C for 72 hours and then harvested at 2600 x g for 5 minutes. The pellet was washed with PBS without Ca or Mg and centrifuged at 1200 x g for 10 minutes. The cells were lysed by sonication in PBS containing a cocktail of protease inhibitors. The solubilized protein preparation was purified by metal chelate chromatography according the AI BioTech’s standard operating procedures. The purity of the recombinant proteins, as determined by HPLC, was 92.5% and the concentration was determined by BCA protein assay.

For the ELISA, clear 96-well EIA (Corning) plates were coated overnight at 4°C with 34.5 ng of the purified N per well. The plates were then blocked in PBST with 3% nonfat dry milk (BD Biosciences) and 3% goat serum (Corning) for 2 h at 37°C, and washed with PBST. Twenty-eight days post-challenge, the sera were heat treated for 30 m at 56°C to inactive virus. Sera were diluted in half-log dilutions in blocking buffer, starting at 1:50. The diluted sera were added to the wells and the plates were incubated at 37°C for 1 h. The plates were then washed with PBST, and probed for 1 h at 37°C with a 1:1000 dilution HRP-conjugated goat anti-mouse antibody (Abcam), and then washed in PBST. The HRP was detected with TMB (SeraCare Inc.) and the plates were read at 450 nm absorbance.

### Statistics

Weight loss significance was determined using two-way ANOVA with the Bonferroni’s *post hoc* correction. Survival statistics utilized the log-rank test. Statistical significance of CCHF_VLP_ total IgG/avidity ELISA and neutralization data were assessed using one way (Tukey’s *post hoc* correction) and two way (Sidak’s *post hoc* correction) ANOVA respectively. Isotype ELISA data analysis was also performed using a two way ANOVA with Sidak’s *post hoc* correction. Isotype ratio analysis was performed using a Student’s t-test. Significance levels were set at a *p* value less than 0.05. All analyses were performed using GraphPad Prism v.6.

## Results

### Expression of a gene-optimized CCHFV-M DNA vaccine construct

**Fig 1:**
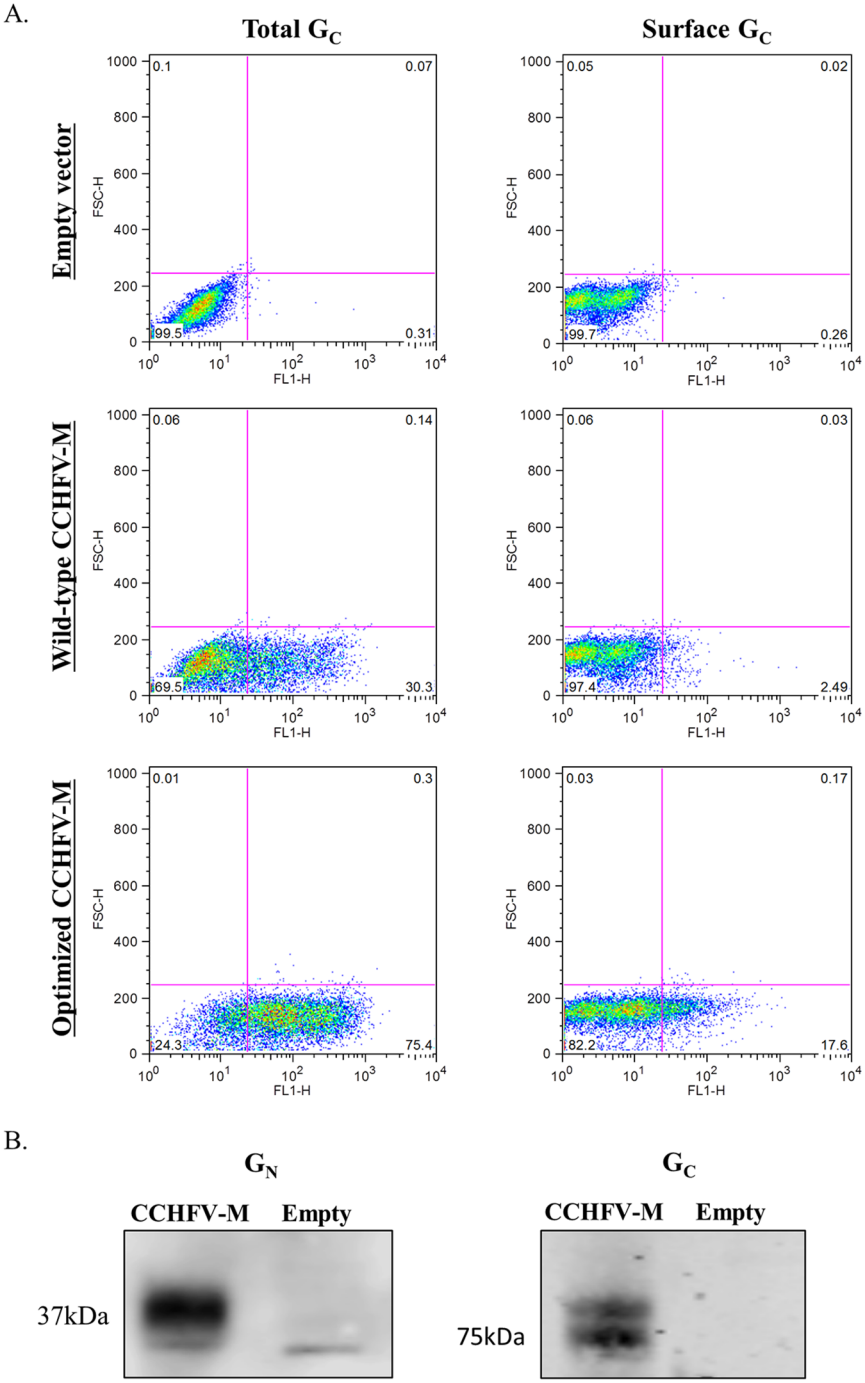
*In vitro* expression of the glycoprotein genes from the CCHFV-M DNA vaccine plasmid. A) The total (permeabilized cells) and surface presence (non-permeabilized cells) of G_C_ was examined 44 h after transfection of COS-7 cells with wild-type CCHFV-M, optimized CCHFV-M, or empty vector, the maximum expression was seen at 250 ng of each plasmid (shown). B) *In vitro* expression by Western blot of G_N_ (37 kDa) and G_C_ (75 kDa) in COS-7 cells 44 h after transfection of CCHFV-M or empty vector, 250 ng of each plasmid.

In earlier studies we found that a DNA vaccine expressing the M-segment ORF of CCHFV did not consistently elicit neutralizing antibodies in vaccinated mice [24]. In studies with a DNA vaccine for Venezuelan equine encephalitis virus, we found that gene optimization could lead to a dramatic improvement in expression and immunogenicity [30]. Consequently, we generated a new construct in which the CCHFV M-segment gene was optimized to reflect the codon bias of humans and to remove known elements that impact mRNA stability and expression. Using flow cytometry, we showed that a monoclonal antibody to CCHFV G_C_ detected viral protein both on the surface of transfected non-permeabilized COS-7 cells and within permeabilized cells (Fig 1A). Expression levels were observed to be dose dependent (S1 Fig); there were 7-fold and 2.5-fold increases of cell surface G_C_ and total G_C_, respectively, between the optimized CCHFV-M vaccine relative to the original wild-type CCHFV-M vaccine. Furthermore, we confirmed expression of both CCHFV glycoprotein genes by Western blot using a rabbit polyclonal antibody to detect G_N_ and a mouse monoclonal antibody to detect G_C_ (Fig 1B).

### Vaccination and challenge of IFNAR^-/-^, and IS mice

To compare the protective efficacy of the optimized CCHFV-M DNA vaccine in two lethal mouse models, we vaccinated groups of 10 IFNAR^-/-^ (C57BL/6 background) mice or immunocompetent C57BL/6 mice three times at 3-week intervals by IM-EP with 25 μg of either the CCHFV-M vaccine or empty pWRG7077 DNA plasmid vector. Blood collection was performed prior to each vaccination to measure antibody responses. Four weeks after the third vaccination, mice were challenged by IP injection with 100 PFU of CCHFV. We previously performed a 99% lethal dose study in IFNAR^-/-^ mice (C57BL/6 background) challenging IP with 10 PFU, 100 PFU, 1000 PFU, and 10,000 PFU (S2 Fig). All challenge doses resulted in 100% lethality between 4 and 5 days and the survival curves of mice in all dosage groups higher than 10 PFU did not differ significantly. These results are similar to those reported previously [16]. We chose the IP route for challenge as IP is a surrogate for intravenous infection, and IP challenge of IFNAR^-/-^ mice was previously found to result in a more rapid onset of disease than challenge by subcutaneous, intranasal, or intramuscular routes in both low dose and high dose challenges [16]; thus, the IP route should provide a stringent test of the vaccine’s efficacy. For the IS model, the vaccinated C57BL/6 mice were immunosuppressed by treatment with an antibody to the IFN-α/β receptor (MAb-5A3) 1 day before and 1 day after challenge by the IP route as described in Methods. The dose and frequency of the MAb-5A3 was empirically determined to ensure >90% lethality.

**Fig 2:**
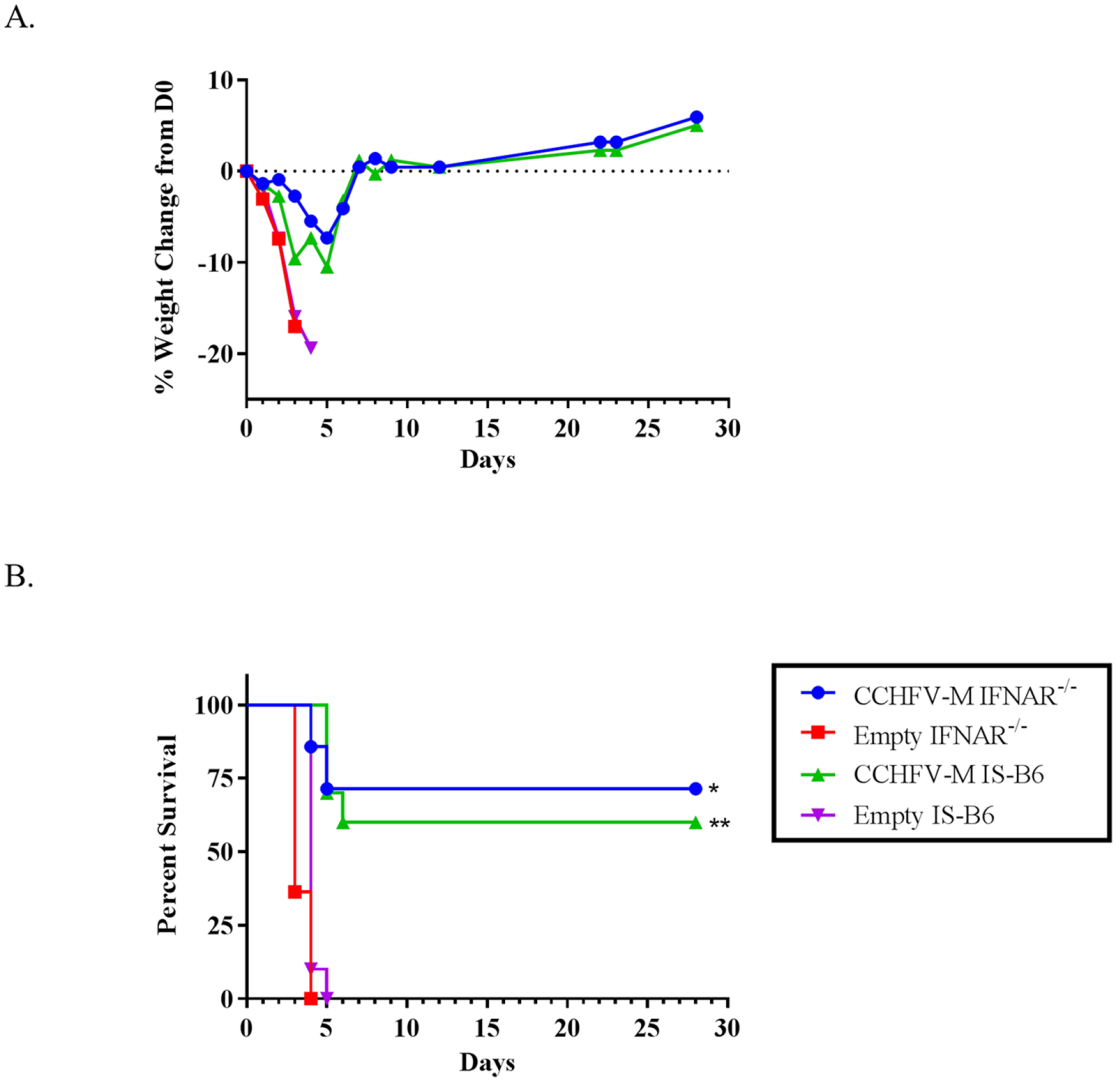
The CCHFV-M DNA vaccination provided protection in both mouse infection models. Group weight (A) and survival (B) following CCHFV challenge with 100 PFU by the IP route, vaccinated C57BL/6 mice were transiently immunosuppressed prior to challenge (IS-B6). CCHFV-M vaccinated group in each mouse strain compared to empty vector vaccinated group in the same strain. Log-rank test, confidence intervals were set to 95%, *p = 0.0002, **p<0.0001.

Following challenge, group weights (Fig 2A) were obtained daily. All of the mice in both empty vector control groups displayed dramatic weight loss and died or were euthanized between days 3 and 5 post-infection (Fig 2A and 2B). Both the CCHFV-M-vaccinated IFNAR^-/-^ and IS mice lost between 5–10% of their group weights by day 6, but survivors returned to their starting weights by day 7 (Fig 2B) and had no visible signs of illness (lethargy, ruffling). The CCHFV-M mice that succumbed to the virus had similar clinical signs as the control animals. Three mice in the CCHFV-M DNA vaccinated IFNAR^-/-^ group died during manipulations three weeks following the final vaccination and prior to challenge. Two out of seven (29%) CCHFV-M DNA-vaccinated IFNAR^-/-^ mice died between days 4 and 5 post-infection, and four out of 10 (40%) CCHFV-M vaccinated C57BL/6 mice died or were euthanized on day 5 post-infection. There was no significant difference between the survival rates of CCHFV-M-vaccinated mice in the two mouse models, although there was a significant difference in both models as compared to mice that were vaccinated with empty vector (Fig 2B).

### Comparison of antibody responses of IFNAR^-/-^ and immunocompetent C57BL/6 mice

**Fig 3:**
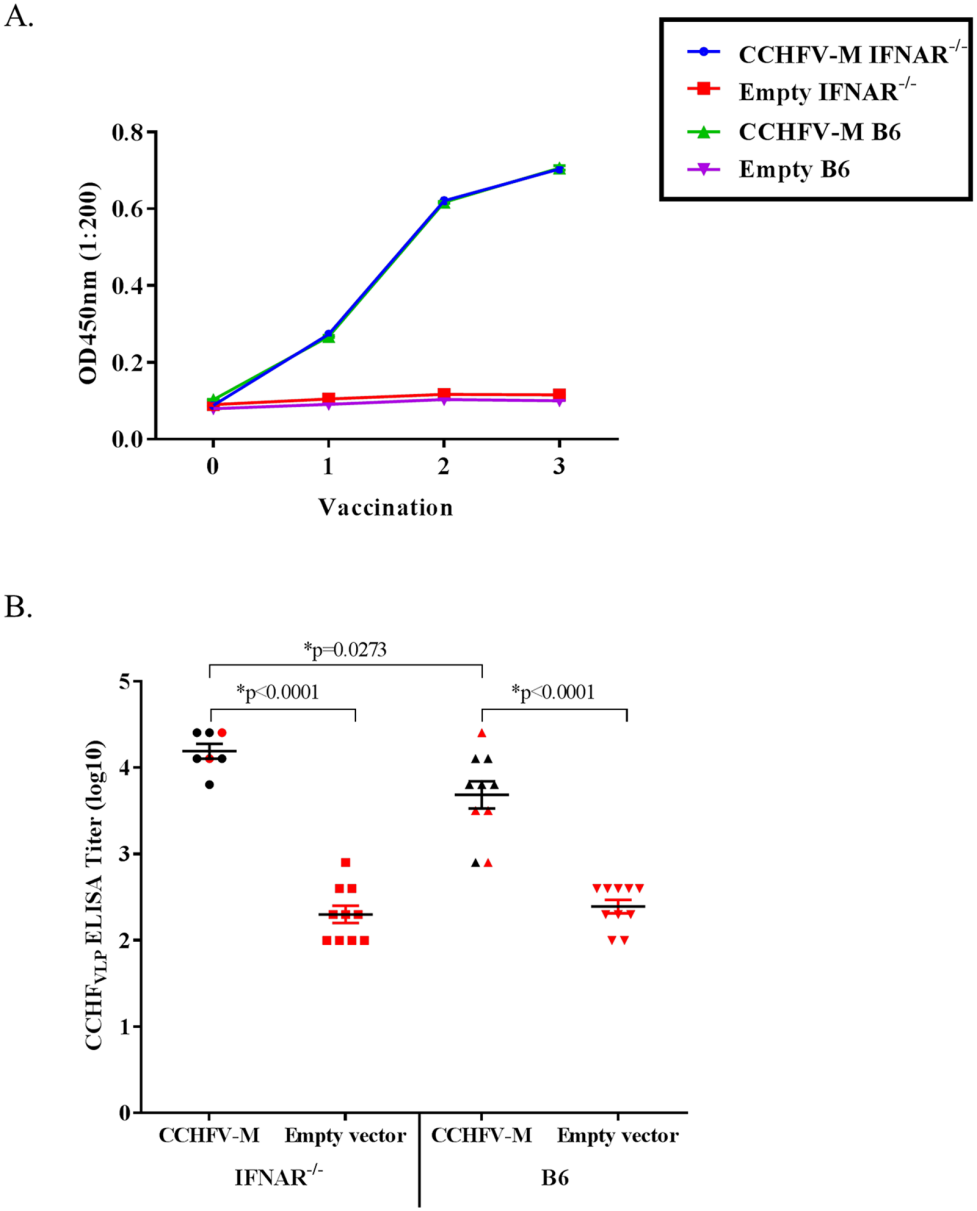
CCHFV-specific IgG ELISA titers increase following each vaccination. The CCHFV-specific ELISA titers following three vaccinations are similar in IFNAR^-/-^ and WT C578BL/6 mice before challenge. A) Mouse sera were pooled in each vaccination group and the CCHFV-specific IgG antibodies were measured by CCHF_VLP_ ELISA following each vaccination with the optimized CCHFV-M vaccine. Vaccinations were performed at weeks 0, 3, and 6. B) The CCHFV IgG ELISA titers for individual mice 1 week prior to challenge; mice that died after CCHFV challenge are shown in red. *One-way ANOVA, confidence intervals were set to 95%.

Total CCHFV glycoprotein-specific antibodies were measured after each vaccination by ELISA using a CCHF_VLP_ antigen. All of the CCHFV-M-vaccinated mice developed CCHFV-specific antibody responses following three vaccinations. The kinetics of the antibody responses were the same for the immune competent C57BL/6 mice and the IFNAR^-/-^ mice; i.e., both mouse strains displayed detectable antibody responses after the first vaccination, large increases after the second vaccination, and a smaller increase after the third vaccination (Fig 3A). Although significantly higher total antibody titers were measured for individual IFNAR^-/-^ mice vaccinated with CCHFV-M as compared to C57BL/6 CCHFV-M vaccinated mice, there was no correlation with ELISA titer and survival after challenge for either mouse strain (Fig 3B).

**Fig 4:**
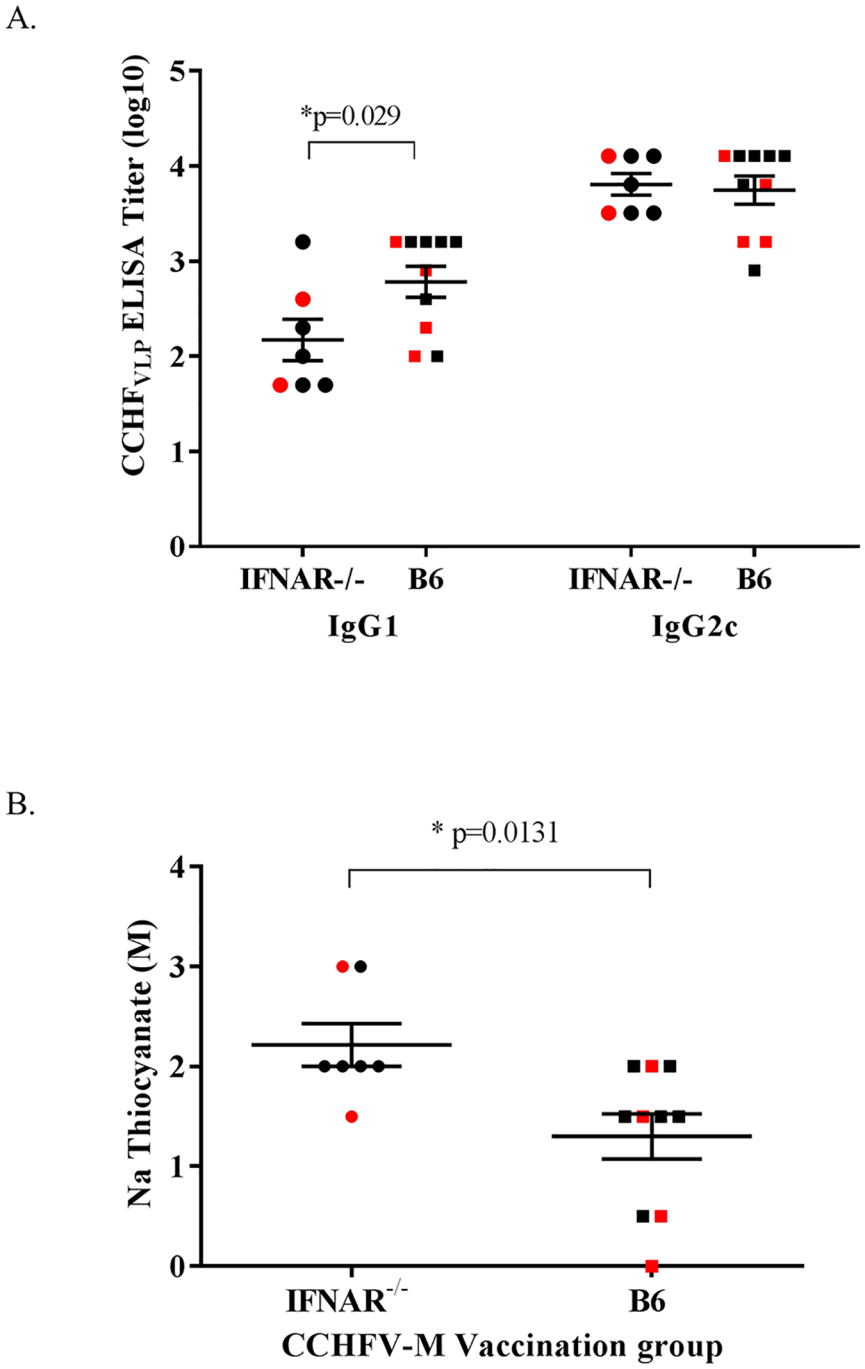
The majority of the CCHFV-M vaccinated mice developed both IgG1 and IgG2c responses following three vaccinations. Prior to challenge, CCHFV-specific antibody isotypes and the antibody avidity were examined by ELISA against the CCHF_VLP_. A) The CCHFV-specific IgG1 and IgG2c response in individual mice following three vaccinations. Pooled sera from IFNAR^-/-^ and WT C57BL/6 mice vaccinated with empty vector were tested concurrently and had no detectable signal. B) The avidity of the CCHFV-specific antibody response in vaccinated mice following three vaccinations was measured. For (A) and (B) mice that died after CCHFV challenge are shown in red. *Two-way ANOVA, confidence intervals were set to 95%.

As an indirect measure of the Th1 vs Th2 response to the CCHFV-M DNA vaccine, we performed IgG2c vs IgG1-specific ELISAs on samples collected 2 weeks after the final vaccination. Both strains of mice had higher IgG2c then IgG1 responses (Fig 4A) indicating a predominant Th1 response, which is consistent with previous trends seen in mice vaccinated by IM-EP or needle delivery [30]. All of the CCHFV-M vaccinated mice developed measureable IgG2c responses and there was no significant difference between titers observed in the two mouse strains. There was a significant difference in the IgG1 response between the IFNAR^-/-^ group and the C57BL/6 group, with 42.8% (3 out of 7) of the IFNAR^-/-^ mice having detectable IgG1, and 80% (8 out of 10) of the WT C57BL/6 mice having detectable IgG1. The ratio of IgG2c to IgG1 was significantly greater in the IFNAR^-/-^ mice than in the WT C57BL/6, with a ratio of 1.85 and 1.39 respectively (p = 0.0422), indicating that overall the immunocompetent mice may have a more balanced response than the IFNAR^-/-^ mice. We also confirmed the ability of our CCHFV-M DNA vaccine to induce affinity maturated B cell responses by avidity ELISA (Fig 4C). Estimated avidity of the CCHFV-specific antibodies in the CCHFV-M vaccinated IFNAR^-/-^ group was significantly higher than the WT C57BL/6 group, however, this did not correlate to a higher survival in the IFNAR^-/-^ mice.

**Fig 5:**
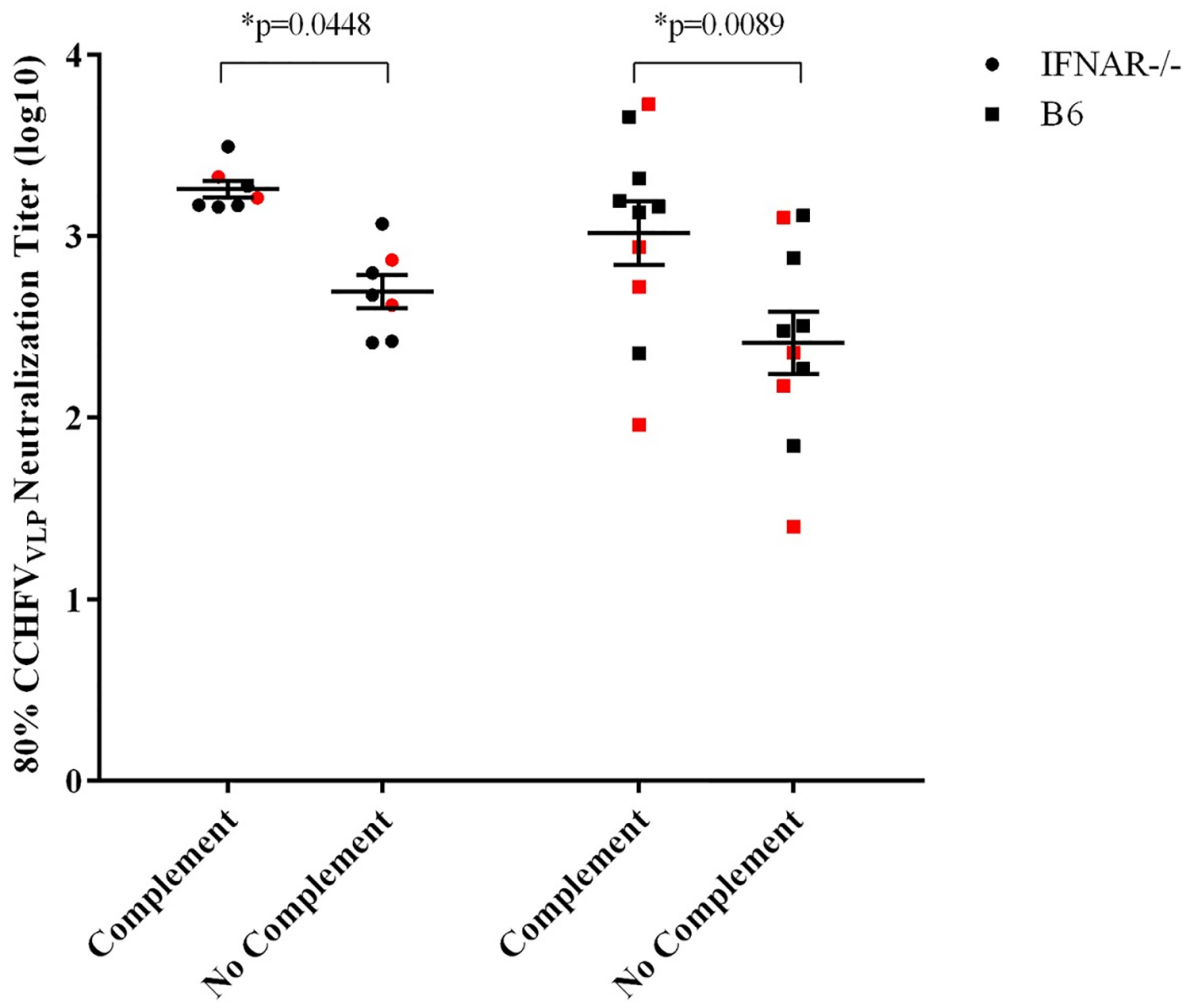
CCHFV-M vaccinated mice developed CCHFV-specific neutralizing antibodies following three vaccinations, and the response was enhanced with the addition of complement. Prior to challenge, neutralization titers were measured against the CCHF_VLP_, with and without complement. Mice that died following challenge are highlighted in red. *Two-way ANOVA confidence intervals were set to 95%.

To assess the neutralizing antibody responses to the CCHFV-M DNA vaccine we used a CCHF_VLP_ neutralization assay similar to that used in earlier studies comparing neutralizing and non-neutralizing monoclonal antibodies [31]. We found that this assay provided similar results as live virus neutralization when tested with a panel of CCHFV-specific monoclonal antibodies (S3 Fig). All of the CCHFV-M DNA-vaccinated IFNAR^-/-^ mice and 90% of the C57BL/6 mice developed neutralizing antibodies to CCHFV. Although there was no significant difference in the group titers of the two mouse strains, the IFNAR^-/-^ mice all had consistent antibody responses as compared to one another whereas there was a wide range of responses among the C57BL/6 mice. There was no significant difference in the CCHFV-specific neutralizing response between the survivors and the non-survivors in either mouse model. For both mouse strains, the addition of complement significantly increased the neutralizing antibody titers (Fig 5).

Our CCHFV-M DNA vaccine was immunogenic in both mouse models, as all of the vaccinated mice developed antibody responses to CCHFV. There was no clear correlation between the humoral response to the vaccine and survival after CCHFV challenge in either IFNAR^-/-^ or WT C57BL/6 mice. Mice that developed a higher CCHFV-specific antibody response, a higher IgG1 response, higher neutralizing antibody titers, or higher antibody avidity did not have a corresponding increase in survival.

### Seroconversion of vaccinated mice following challenge

**Fig 6:**
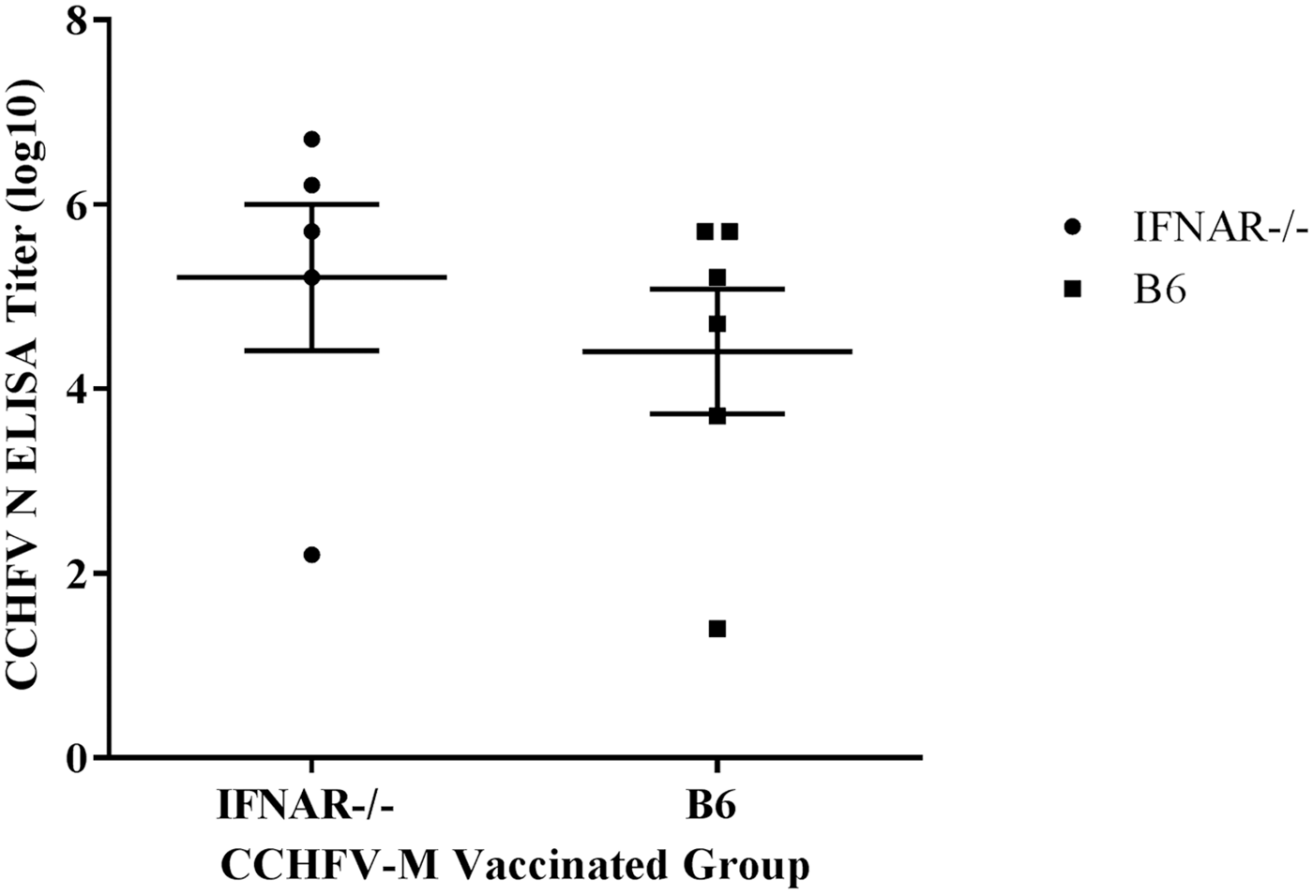
N-specific antibodies in post-challenge sera of CCHFV-M vaccinated mice. Sera from vaccinated mice that survived CCHFV challenge (28 days post-challenge) were examined by ELISA for antibodies against N.

To determine if mice that survived CCHFV challenge had been infected we measured antibodies to CCHFV N in sera collected from mice four weeks after challenge. CCHFV N was not encoded in our vaccine construct, so the presence of anti-N antibodies would suggest viral replication. All of the IFNAR-/- mice and all but one mouse of the IS C57BL/6 group had detectable antibodies to CCHFV N, indicating that the vaccine did not provide sterile immunity (Fig 6). There was no difference in the CCHFV-N antibody response between the IFNAR^-/-^ and the IS mice, and the one IS C57BL/6 mouse that did not have detectable anti-N antibodies did not have a higher antibody response to the CCHFV-M vaccine than mice that succumbed to the infection.

## Discussion

We generated a DNA vaccine construct encoding the M-segment ORF of CCHFV, which was optimized for expression in mammalian cells. We compared and quantified both the humoral response and protective efficacy of our optimized DNA vaccine in two murine challenge models (IFNAR ^-/-^ and IS) with the same genetic background (C57BL/6). We found that the optimized CCHFV-M DNA vaccine delivered by IM-EP was highly immunogenic, with 100% of IFNAR^-/-^ vaccinated mice and 90% of C57BL/6 mice developing CCHFV-specific immune responses, including neutralizing antibodies. The single mouse in the IS model that failed to produce neutralizing antibodies did develop G_N_/G_C_-specific antibodies, albeit at low levels. The immunocompetent C57BL/6 mice developed a more balanced IgG2c/IgG1 response than the IFNAR^-/-^ mice, which may be due to cytokine signaling differences in the immunocompetent mice. In both mouse models, the CCHFV-specific IgG ELISA titers of vaccinated mice significantly increased between the second and third boosting vaccinations. Because we did not test additional vaccinations, we do not know if we reached the maximum response possible.

As we were preparing this manuscript, another study reported DNA vaccination of IFNAR^-/-^ mice with an A129 background using separate plasmids expressing CCHFV G_N_, G_C_, or N genes, each tethered to a ubiquitin coding sequence [23]. In general, our findings of the humoral immune response to DNA vaccination are in agreement with those in the other DNA vaccine report; however, numerous differences between the two studies make it difficult to directly compare results. For example, our vaccine expresses the complete M-segment ORF of CCHFV, whereas in the other study a mixture of plasmids was used, which included an N construct along with two constructs encoding the individual glycoprotein genes, from which the mucin-like domain and GP38 coding regions were deleted. Also, our vaccine does not express either the N gene or the ubiquitin sequence. The ubiquitin was intended to broaden the cell-mediated immune response, but it is difficult to determine if it did due to the small number of mice used and the limited sample volumes that precluded a comprehensive assessment of cell mediated immunity. In addition to differences in the DNA vaccine constructs and the differing genetic backgrounds of the IFNAR^-/-^ mice, the studies differed in that we used a lower dose of DNA and a different delivery method (IM-EP vs intradermal EP) and compared the IFNAR^-/-^ mouse model responses to antibody responses of immunocompetent mice.

In our studies, we could not identify a correlate of protective immunity in either mouse model. While we were able to elicit specific anti-CCHFV antibody responses in all vaccinated mice, post-challenge seroconversion ELISA results revealed that the vaccine was not able to prevent viral replication in the majority of the CCHFV-M DNA-vaccinated mice. These results are similar to those reported previously with a MVA-GP vaccine, and in the recent CCHFV DNA vaccine study [20]. A comparative study between vaccinated and control mice to determine if vaccination reduces viral burden during the acute stage of disease will be included in future studies. In addition, it is possible that we did not achieve sufficient expression levels of the proteins to elicit full protective immunity in mice; therefore, we will examine whether a higher vaccine dose and/or improved expression will increase the immunogenicity and durability of the DNA vaccine *in vivo*.

Also consistent with the MVA-GP and the DNA vaccine studies, we found no direct correlation between the humoral response(s) and survival in CCHFV-M DNA-vaccinated mice [20] suggesting that anti-CCHFV glycoprotein antibodies alone elicited by these vaccines are not sufficient for protection against viral challenge. This is in agreement with results of an earlier study demonstrating that the passive transfer of serum antibodies from MVA-GP vaccinated mice into a naïve host did not confer protection to CCHFV challenge [22]. MVA-GP vaccine studies further suggested, through adoptive transfer of T-cells and passive sera transfer studies, that both the cellular and humoral responses to the MVA-based CCHFV vaccine were necessary to provide protection, as determined by a statistically significant delay in time to death. Contrary to this, earlier passive transfer studies with monoclonal antibodies directed against CCHFV G_N_/G_C_ show that individual neutralizing and non-neutralizing antibodies alone can provide 100% protection both before and after challenge in suckling mice [35]. However, monoclonal antibody studies have not been reported to confirm that this protection holds true in an adult mouse model. As we have not yet assessed cell-mediated immune responses to our DNA vaccine, we cannot eliminate the necessity for inclusion of additional immunogens, such as N, to elicit T cell responses, although the immune response elicited by the MVA-GP vaccine was fully protective, whereas MVA-N vaccine was unable to protect.

We show in this study that immune competent mice can be used to evaluate CCHFV vaccines and protective efficacy can be examined by transient inhibition of IFN-I using MAb-5A3 proximal to the time of challenge. IFN-α/β signaling is critical for the generation of potent adaptive immune responses, for example by promoting antigen-presenting cell maturation, driving the T cell, and subsequent B cell, response [36, 37]. IFN-α/β also amplifies B cell receptor sensitivity, boosting the ability of naïve B cells to produce antibodies upon antigen recognition [38]. Furthermore, IFN-α/β signaling promotes the generation of memory T and B cell pools. Although we did not observe significant differences either in antibody responses or protective immunity in the IFNAR^-/-^ vs the IS models with our CCHFV M DNA vaccine, the ability to vaccinate immune intact mice might be advantageous for other DNA vaccine approaches or for other types of CCHFV vaccines. For example, in the same study where the mixed CCHFV DNA vaccine plasmids were tested, a transcriptionally-competent CCHF VLP (tcVLP) vaccine was given alone or in a prime-boost regimen with the DNA. The IFNAR^-/-^ mice vaccinated with the tcVLP or the prime-boost were less protected than the DNA vaccine alone [23]. The authors concluded that a type I IFN responses may be required for the development of a protective immune response against the tcVLP vaccine. Therefore, the ability to study CCHFV vaccines in immune intact mice and then testing protective efficacy by disrupting IFN signaling only at the time of challenge might have important advantages over the IFNAR^-/-^ CCHFV vaccination model, particularly when T cell responses are critical for protection [39, 40].

In summary, here we show that a novel CCHFV M-segment DNA vaccine can elicit protective immune responses to CCHFV challenge in two lethal mouse models of CCHF. The exact mechanism of protection remains unclear, but it is evident that a DNA vaccine encoding the CCHFV M-segment ORF can generate protective immunity. It remains to be seen if this vaccine can provide cross-protective immunity to more genetically distant CCHFV strains. Overall, our results provide further insight into the protective capabilities of a CCHFV DNA vaccine and will help in the development of a more rationally tailored CCHFV vaccine.

## Supporting information

S1 Fig Dose curve of *in vitro* expression of the glycoprotein genes from the CCHFV-M DNA vaccine plasmid. A) The total (permeabilized cells) and (B) surface presence (non-permeabilized cells) of G_C_ was examined 44 h after transfection of COS-7 cells with wild-type CCHFV-M (CCHFV-M WT) and optimized CCHFV-M (CCHFV-M opt) with a dose curve of 50–250 ng of each plasmid.
(TIF)

S2 Fig 99% lethal dose (LD99) study for the IP route in IFNAR^-/-^ mice. Mice received 10 PFU to 10,000 PFU of CCHFV IbAr 10200 IP to determine the LD_99_. A) Survival. B) Group weights.
(TIF)

S3 Fig Comparison of neutralization of CCHF VLPs versus live CCHFV by monoclonal antibodies against the G_N_ or G_C_ glycoproteins. CCHF VLPs were mixed with indicated dilutions of monoclonal antibodies, and added to SW13 target cells for 24 hrs prior to measurement of luciferase activity. Fifty-percent neutralization titers with the VLPs are reported (black bars). Included is a comparison to historical plaque reduction neutralization data performed with live virus (grey bars). As with VLPs, 50% neutralization titers are shown.
(TIF)
