# Supplementary figures and images for: A DNA vaccine for Crimean-Congo hemorrhagic fever protects against disease and death in two lethal mouse models
Source: PLoS Negl Trop Dis. 2017 Sep 18;11(9):e0005908. doi: 10.1371/journal.pntd.0005908 (PMC5619839; doi:10.1371/journal.pntd.0005908)

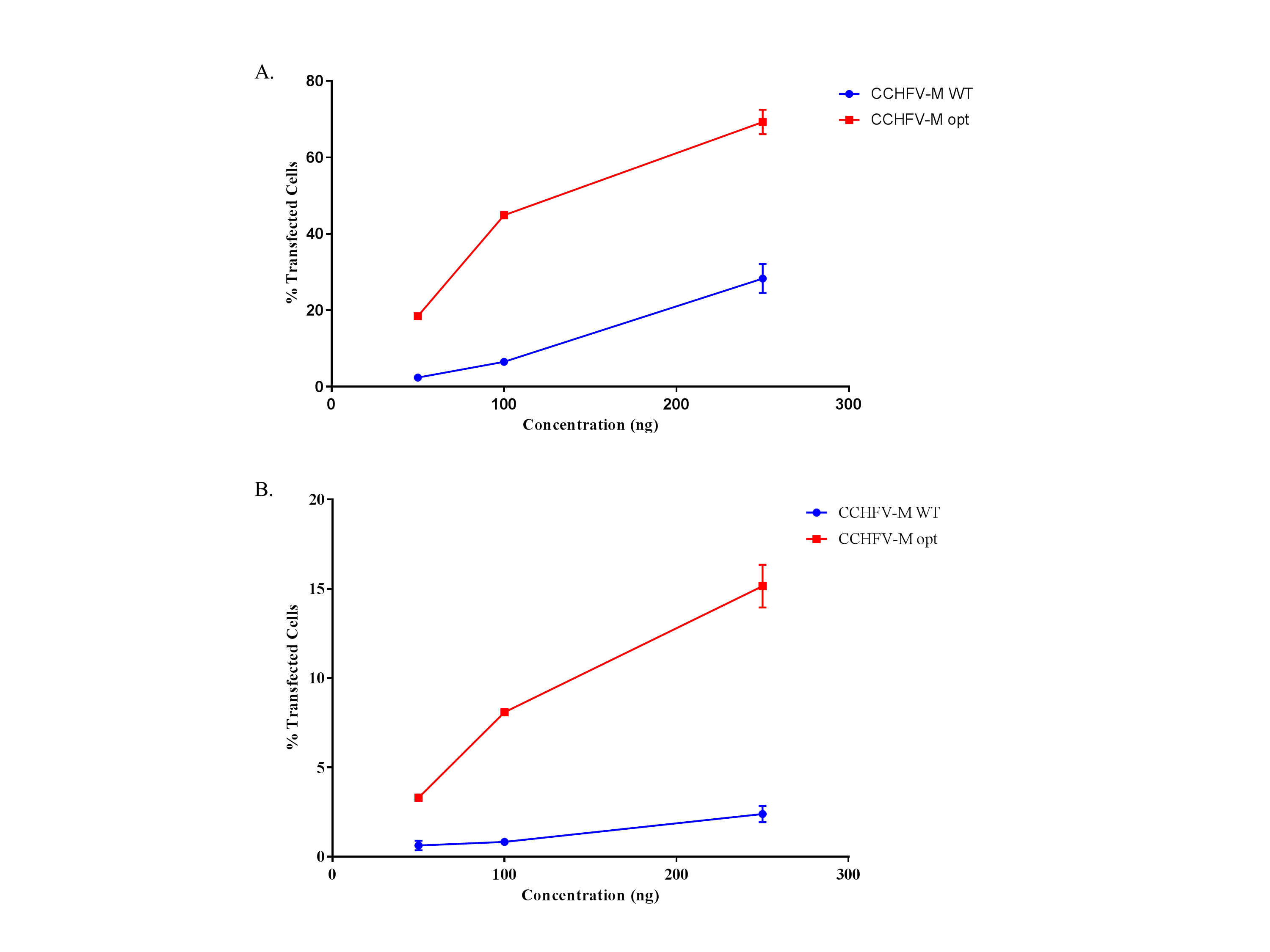

Supplement: S1 Fig — A) The total (permeabilized cells) and (B) surface presence (non-permeabilized cells) of GC was examined 44 h after transfection of COS-7 cells with wild-type CCHFV-M (CCHFV-M WT) and optimized CCHFV-M (CCHFV-M opt) with a dose curve of 50–250 ng of each plasmid. (TIF) [file pntd.0005908.s001.tif]

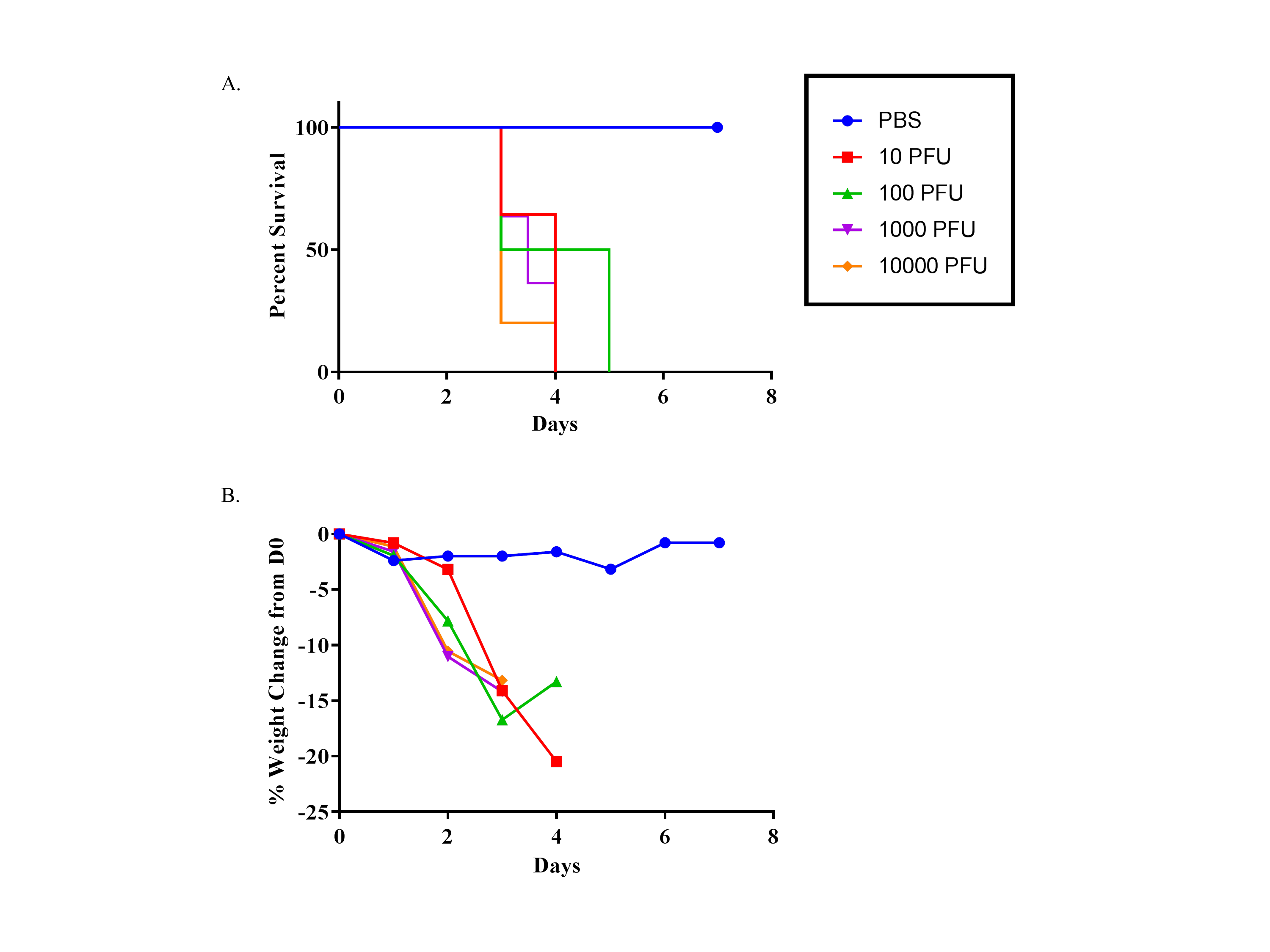

Supplement: S2 Fig — Mice received 10 PFU to 10,000 PFU of CCHFV IbAr 10200 IP to determine the LD99. A) Survival. B) Group weights. (TIF) [file pntd.0005908.s002.tif]

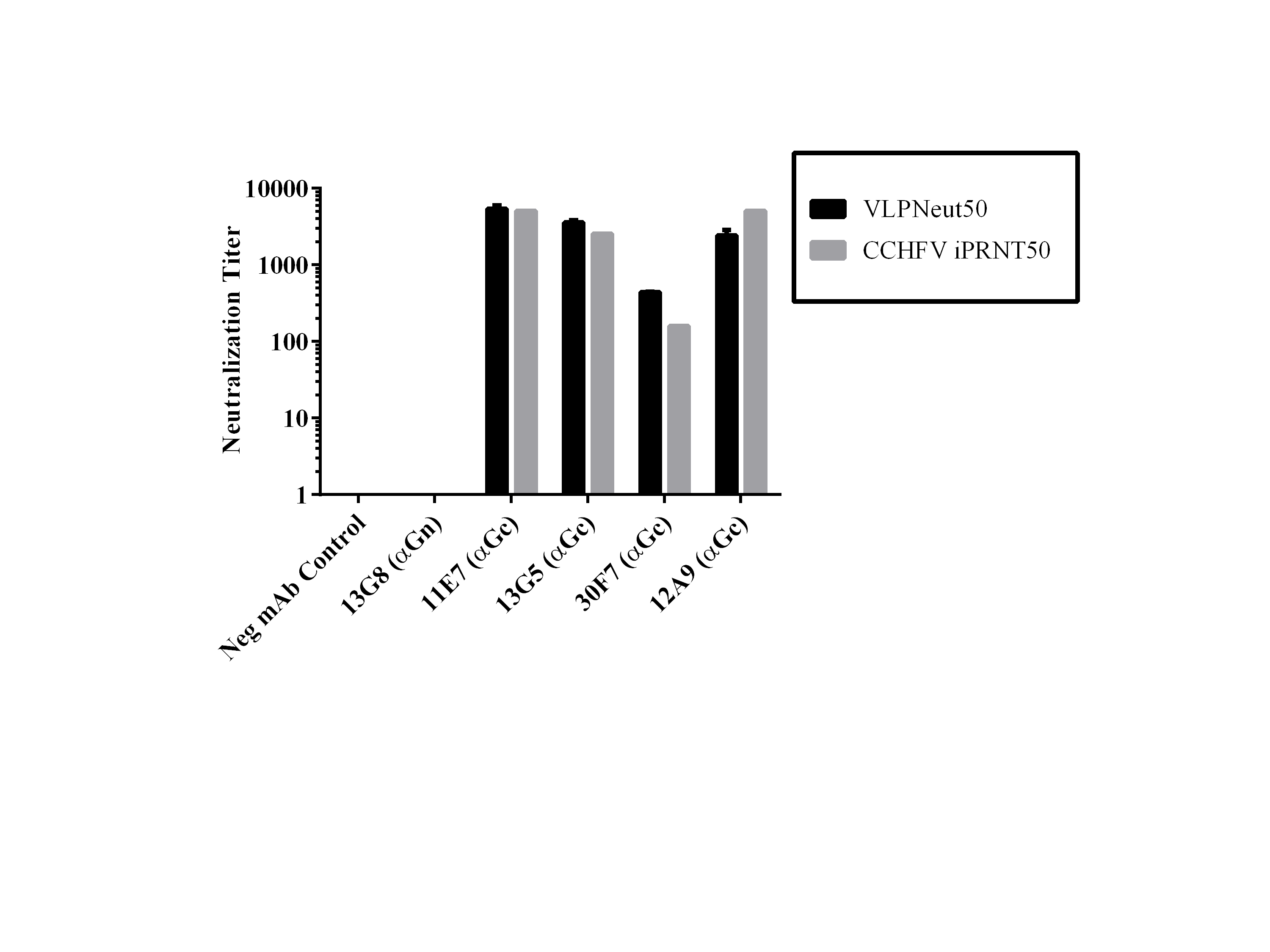

Supplement: S3 Fig — CCHF VLPs were mixed with indicated dilutions of monoclonal antibodies, and added to SW13 target cells for 24 hrs prior to measurement of luciferase activity. Fifty-percent neutralization titers with the VLPs are reported (black bars). Included is a comparison to historical plaque reduction neutralization data performed with live virus (grey bars). As with VLPs, 50% neutralization titers are shown. (TIF) [file pntd.0005908.s003.tif]
